# Ketogenic Diet in Epileptic Encephalopathies

**DOI:** 10.1155/2013/652052

**Published:** 2013-07-10

**Authors:** Suvasini Sharma, Manjari Tripathi

**Affiliations:** ^1^Department of Pediatrics, Lady Hardinge Medical College and Associated Kalawati Saran Children's Hospital, New Delhi 110001, India; ^2^Department of Neurology, Neurosciences Centre, All India Institute of Medical Sciences, New Delhi 110029, India

## Abstract

The ketogenic diet is a medically supervised high-fat, low-carbohydrate diet that has been found useful in patients with refractory epilepsy. It has been shown to be effective in treating multiple seizure types and epilepsy syndromes. In this paper, we review the use of the ketogenic diet in epileptic encephalopathies such as Ohtahara syndrome, West syndrome, Dravet syndrome, epilepsy with myoclonic atonic seizures, and Lennox-Gastaut syndrome.

## 1. Introduction

Epileptic encephalopathies are disorders in which the presence of frequent and difficult-to-control seizures along with unremitting interictal epileptiform activity contributes to progressive neurodevelopmental deterioration [1]. These have been described based on their electroclinical features (age of onset, seizure type, and EEG pattern). These include Ohtahara syndrome, early myoclonic encephalopathy, epilepsy of infancy with migrating focal seizures, West syndrome, Dravet syndrome, Lennox-Gastaut syndrome, epilepsy with myoclonic atonic seizures, and Landau-Kleffner syndrome. These epilepsies are often described as “catastrophic epilepsies” as they are difficult to treat despite the use of multiple anticonvulsant drugs. They are often associated with a poor cognitive and neurodevelopmental outcome. 

The ketogenic diet (KD) is a high-fat, low-carbohydrate, and restricted protein diet that has been found useful in patients with refractory epilepsy [2]. One systematic review showed complete cessation of seizures in 16% of children, greater than 90% reduction in 32%, and greater than 50% reduction in 56% with the use of the KD [3]. The KD has also been found effective in children with refractory epilepsy in a randomized controlled trial [4]. A recent Cochrane review concluded that the ketogenic diet results in short- to medium-term benefits in seizure control, the effects of which are comparable to modern antiepileptic drugs [5].

The modified Atkins diet is a less restrictive alternative to KD [6]. In this diet, carbohydrates are restricted to 10 to 20 grams/day. Fats are actively encouraged, and proteins can be given unlimited. A number of studies in the last few years have shown a similar efficacy to the KD [7–10]. Approximately, half of the patients have a better than 50% improvement in seizures with this diet. 

The KD has been found particularly useful in some epileptic encephalopathies of young children such as West syndrome and epilepsy with myoclonic atonic seizures. In this paper, we review the use of the ketogenic diet in various epileptic encephalopathies.

## 2. Ohtahara Syndrome

Ohtahara syndrome has an onset within the first few days or weeks of life with predominant tonic spasms that may be single or occur in clusters [11]. These occur both in sleep and waking states. The EEG shows a burst suppression pattern that is present both in the sleep and awake states. The most frequently association reported is brain malformations, for example, cortical dysplasia, hemimegalencephaly, and Aicardi syndrome. 

There have been isolated case reports of the use of the KD in these syndromes. Ishii et al. reported a male infant with Ohtahara syndrome who failed serial trials of high-dose pyridoxal phosphate; antiepileptic drugs; sodium valproate, phenobarbital, clonazepam, and clobazam in various doses and combination; ACTH; TRH; and gamma globulin. Then, the KD diet was tried and found effective in controlling the seizures [12]. 

## 3. Early Myoclonic Encephalopathy

Early myoclonic encephalopathy also presents in the first few days of life. These infants have mixed seizures: erratic or fragmentary myoclonus, focal seizures, and tonic seizures [13]. The EEG shows a burst suppression pattern that is more prominent during sleep and may disappear in the awake state. The common etiologies are inborn errors of metabolism such as nonketotic hyperglycinemia, organic acidemias, Menkes disease, and Zellweger syndrome. 

Cusmai et al. reported the use of the KD in 3 infants with early myoclonic encephalopathy secondary to nonketotic hyperglycinemia [14]. KD was added to the continuing antiepileptic drug regimen. In two patients, there was >50% reduction in seizures, while in the third, only a mild reduction in seizures was noted. Improved alertness was seen in one child. No significant adverse effects were seen.

## 4. Epilepsy of Infancy with Migrating Focal Seizures

This condition, earlier known as migrating partial seizures in infancy, is a rare, age-specific epileptic encephalopathy with a malignant course. It is characterized by onset in the first 6 months of age, after a normal early development, of nearly continuous multifocal partial seizures arising independently and sequentially from both hemispheres, progression through a period of intractable seizures, subsequent neurologic deterioration or arrest with complete loss of both cognitive and motor abilities, and, in most children, progressive decline of head circumference percentile [15].

There is limited data on the use of the KD in this syndrome. Five patients in different case series did not show improvement [16–18]. Thammongkol et al. reported improvement in 2 patients with >50% response after 3 months on the diet [19].

## 5. West Syndrome

West syndrome is characterized by the triad of infantile spasms, hypsarrhythmia on EEG, and developmental delay. Infantile spasms typically occur between 3 and 12 months of age. Spasms usually occur in clusters comprising tonic contractions of limb and axial muscles and may be flexor, extensor, or mixed. The spasms typically occur in relation to the sleep-wake cycle [20]. The etiology is diverse. Any insult to the developing brain (perinatal brain insult, intrauterine infections, inherited metabolic disorders, neurocutaneous syndromes, brain malformations, or postnatal acquired brain insults (e.g., meningoencephalitis and hypoxic brain injury)) during the critical vulnerable time window can lead to West syndrome. However, in as many as 30% of the affected children, no cause can be identified, and these are known as “cryptogenic”. 

The KD has been shown to be an effective treatment for infantile spasms. Hong et al. reported the use of the KD in 104 infants diagnosed with infantile spasms between 1996 and 2009 [21]. Previous treatment for these patients included a mean of 3.6 anticonvulsants, 71% including corticosteroids or vigabatrin. A better than 50% spasm improvement occurred in 64% at 6 months and 77% after 1-2 years. Thirty-eight (37%) became spasm-free for at least a 6-month period within a median 2.4 months of starting the KD. In addition, 62% reported improvement in development, 35% had EEG improvement, and 29% were able to reduce concurrent anticonvulsants. The authors also reported that older age at the onset of infantile spasms and fewer prior anticonvulsants were more likely to be associated with >90% spasm improvement at 6 months. Eun et al. reported the use of the KD in 43 children with intractable infantile spasms [22]. Overall, the diet achieved spasm freedom in 53.5% of patients and a greater than 90% reduction of seizure frequency in 62.8% of patients. 

The KD may also be considered in new-onset infantile spasms. Kossoff et al. conducted a retrospective study comparing the results of all infants started on the KD versus high-dose ACTH for new-onset infantile spasms [23]. Infants were spasm-free in 8 of 13 (62%) infants treated with the KD within 1 month, compared to 18 of 20 (90%) treated initially with ACTH. When effective, median time to spasm freedom was similar between ACTH and the KD (4.0 versus 6.5 days). Those treated with ACTH were more likely to have a normal EEG at 1 month (53% versus 9%); however, use of the KD led to EEG normalization within 2–5 months in all eight who became spasm-free. Side effects (31% versus 80%) and relapse rate after initial success (12.5% versus 33%) were lower with the KD.

The modified Atkins diet, which is a simpler and easier-to-administer version of the ketogenic diet, has also been found to be effective in children with infantile spasms. In a study of 15 children, aged 6 months to 3 years, with infantile spasms refractory to hormonal therapy and/or vigabatrin, the modified Atkins diet was found to render 6 children (40%) spasm-free with EEG resolution of hypsarrhythmia at 3 months [24]. The diet was well tolerated in these young children.

## 6. Dravet Syndrome

Dravet syndrome is characterized by the occurrence of generalized or unilateral clonic or tonic-clonic seizures, usually triggered by fever, in the first year of life of a previously normal infant [25]. Later, other types of seizures occur, including myoclonus, atypical absences, and partial seizures. Early development is normal and slows in the second year of life. Cognitive decline and behavioral disturbances are frequently noted. Genetic influences predominate as a positive family history is seen in 25%−71% of patients. 

The interictal EEG is usually normal in the first year of life [26]. However, between the second and the fifth years of life, a progressive increase in epileptiform abnormalities with background slowing is seen in more than 50% of the cases. These consist of generalized spike-wave and polyspike-wave discharges, focal and multifocal fast spikes, and polyspikes. Mutations in the SCN1A gene encoding the alpha-1 subunit of the sodium channel are detectable in 70–80% of patients with Dravet syndrome [27].

The KD has been shown to be a good treatment option for Dravet syndrome [28]. Caraballo et al. reported the results of the use of the KD on 20 children with Dravet syndrome. One year after initiating the diet, 13 patients remained on it. Two patients were seizure-free, eight children had a 75–99% decrease in seizures, and the remaining three children had a 50–74% decrease in seizures [29]. In a Korean multicentric study, Kang et al. found similar results in 14 patients with Dravet syndrome [30]. Nabbout et al. reported on the use of the KD in 15 children with Dravet Syndrome who had failed trial of multiple anticonvulsant drugs including stiripentol [31]. At 1 month, 10 patients had a more than 75% decrease in seizure frequency. Efficacy was maintained in eight responders at 3 and 6 months and in six responders at 9 months. Five patients remained on KD for over 12 months, and one was seizure-free. KD was also found to be beneficial on behavior disturbances including hyperactivity. 

## 7. Epilepsy with Myoclonic Atonic Seizures (EMAS)

This epilepsy syndrome was earlier known as myoclonic astatic epilepsy or Doose syndrome. It is characterized by a combination of seizures, including drop attacks and variable psychomotor deterioration beginning in early childhood. It begins between 2 and 5 years of age in a previously healthy child, usually with generalized tonic-clonic seizures. Within a few days or weeks, the patient starts to have falls due to myoclonic seizures [32]. These drop attacks may be very severe, causing injuries. At this point, the child may have tonic-clonic, clonic, absence, and atonic and myoclonic-astatic seizures. Tonic seizures are rare. Cognitive and behavioral deterioration is variable. The MRI brain is normal. 

The KD has been found effective in several series of patients with this disorder [28, 33]. Oguni et al. reviewed the use of KD in 81 patients with EMAS [34]. They found that the KD was the most effective in stopping seizures followed by ACTH and ethosuximide. In their cohort, 55 out of 81 patients achieved complete remission. Fejerman et al. described 11 patients with EMAS treated with the KD [35]. Six had a better than 50% reduction, and four achieved 75–100% seizure reduction.

In a prospective study, Caraballo et al. assessed the effectiveness of the KD in 30 patients with EMAS, 11 of whom had been administered KD [36]. Six remained on the KD after 18 months, two were seizure-free, two had a 75–99% reduction in seizures, and 2 had a 50–75% reduction in seizures. Kilaru and Bergqvist reported the use of KD in 10 children with EMAS [37]. KD was initiated after an average of five anticonvulsant medication trials. Five children became seizure-free. In the remaining children, three had no change in seizure frequency, one developed pancreatitis leading to diet discontinuation, and one had a significant reduction in seizures.

Recently glucose transporter defect has been implicated in EMAS. Mullen et al. showed that 5% of children with EMAS have glucose transporter 1 deficiency with mutations of SLC2A1 [38]. The presence of GLUT1 deficiency suggests that the KD should be introduced early in the treatment with potential beneficial effects for seizure control and neurodevelopmental outcome.

## 8. Lennox-Gastaut Syndrome

Lennox-Gastaut syndrome is one of the most severe childhood-onset epilepsies. It is characterized by mixed seizure types, predominantly tonic seizures. Other seizure types include atypical absence, atonic, and less commonly myoclonic seizures. Generalized tonic-clonic and focal seizures may also occur. The tonic and atonic seizures often lead to drop attacks and falls. The age of onset is between 3 and 10 years. In many patients, there may be a preceding history of infantile spasms. The etiology is variable, and as in West syndrome, the etiology is classified into known (symptomatic) and unknown (cryptogenic) causes. The causes are protean and include malformations of cortical development, sequelae of perinatal insults, inborn errors of metabolism, postnatal meningoencephalitis, and tuberous sclerosis.

Lemmon et al. performed a retrospective review of children with LGS initiated on the KD at the Johns Hopkins Institute from 1994 to 2010 [39]. They also reviewed the literature for cases of LGS treated with the KD and their outcomes. In their center, 71 children with LGS were initiated on the KD. At 6 months, 36 achieved more than 50% seizure reduction, 16 (23%) experienced more than 90% seizure reduction, and 1 (1%) achieved seizure freedom. Results were similar after 12 months. In the historical literature review, the authors found that 88 of 189 (47%) children with LGS had more than 50% seizure reduction after 3 to 36 months of ketogenic diet treatment. Hence the KD seems a good option in this difficult-to-treat epilepsy syndrome.

## 9. Landau-Kleffner Syndrome and Epileptic Encephalopathy with Continuous Spikes and Waves during Sleep

Landau-Kleffner syndrome (LKS) and the syndrome of continuous spikes and waves during slow sleep (CSWS) are closely related epilepsy syndromes. Both are characterized by seizures that are not very frequent or difficult to control, but severe paroxysmal abnormalities on EEG, and consequent language (more prominent in LKS) and cognitive (more prominent in CSWS) deterioration. 

LKS is described as an acquired epileptic aphasia or auditory agnosia, occurring in a previously normal child, with or without clinically apparent seizures [40]. The disorder begins most commonly between the ages of 3 and 8 years. The MRI is normal. Seizures occur in 75% of the patients; these are infrequent and respond well to antiepileptic drugs. The usual seizure types are focal seizures and generalized tonic-clonic seizures. The verbal auditory agnosia may later progress to complete word deafness. The EEG shows mainly posterior temporal spike-wave complexes which increase markedly during NREM sleep. 

Epileptic encephalopathy with continuous spikes and waves during sleep is characterized by a triad of seizures, neuropsychological impairment, and EEG showing continuous spikes and waves during sleep [41]. The seizures are focal or generalized tonic-clonic in type. The cognitive and behavioral disturbances include reduced attention span, hyperactivity, aggressiveness, and severe decrease in the IQ. The EEG shows continuous spike waves with marked sleep activation with anterior predominance.

There is paucity of data on the use of the KD in LKS and CSWS. Bergqvist et al. described three patients with LKS refractory to traditional treatments who were successfully treated with the ketogenic diet [42]. All three patients had lasting improvement of their language, behavior, and seizures for 26, 24, and 12 months, respectively. Nikanorova et al. used the KD in five children aged between eight and 13 years with CSWS refractory to conventional AEDs and steroids [43]. EEG monitoring after 24 months showed remission of CSWS in one child and a mild decrease of spike-wave index in another child. However, the IQ scores were unchanged at the end of the followup.

## 10. Conclusion

The KD is a good treatment option in epileptic encephalopathies especially in West syndrome, Dravet syndrome, and myoclonic astatic epilepsy. Further research is needed to establish the place of KD in the treatment algorithms of these disorders and on the use of less restrictive alternatives such as the modified Atkins diet and the low glycemic index treatment.
